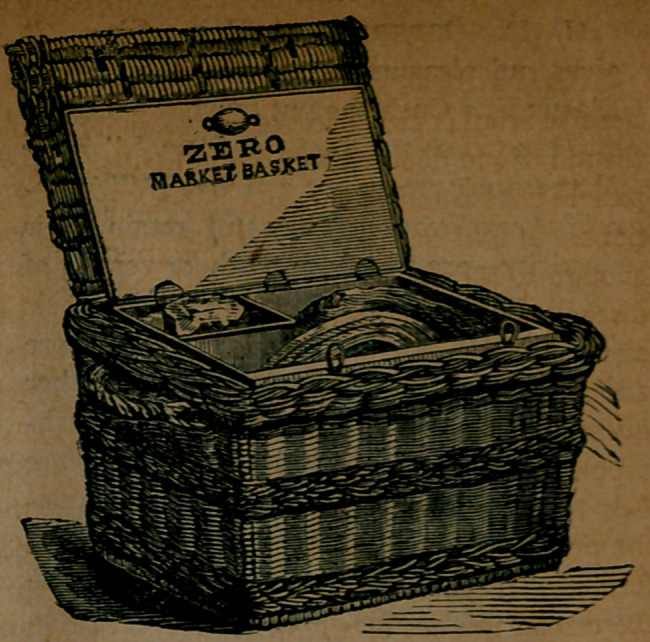# Zero Market Basket

**Published:** 1875-08

**Authors:** 


### THE ZERO MARKET BASKET.

ZERO MARKET BASKET.

Mr. A. M. Lesley, the refrigerator manufacturer, has just perfected another invention as useful in its sphere as any which he has yet offered to his patrons. He calls it the "Zero Market Basket." In its outward appearance it is a basket, and nothing more, made of firm and substantial wicker-work. But on raising the lid we find that the basket contains, or rather is lined with, a galvanized iron box, divided into compartments. One of these contains the ice, and the other may be devoted to meats and other articles of marketing.

People living at a distance can send their basket to market and have it returned with its contents of meat, butter and other articles kept cool and in good order. The double lid fastens closely, entirely excluding dust and flies, and may also be made still more secure by a pad-lock.

The following is the testimony of a well-known citizen:

June 1, '75.

Alex. M. Lesley—*Dear Sir:* I have used your Zero Market Basket during the hot weather with great success, all things keeping perfectly sweet and fresh.

J. D. Prince, *Banker*,
64 *Broadway, and Islip, L.I.*

Excursionists and tourists will find this latest invention of Mr. Lesley a perfect luxury, and having used it once will ever regard it as an indispensable article of their outfit. The sandwiches and "cold cuts are kept cool and fresh, the bottles of cold tea and coffee are palatable and deliciously refreshing, and the wine, provided for the feeble ones of the party, is much more strengthening and more easy to take, than if it was disgustingly warm.

The *Nautical Gazette*, which is recognized as authority on these things, in speaking of the Zero Basket, says, "Used in connection with the line of Newport, Saratoga and Spa coolers, manufactured by Mr. Lesley, the dog-days may be defied by yachtsmen and tourists."

For travellers by railroad, especially when little children are of the party, the Zero Basket is invaluable. Milk and all foods for infants may be kept fresh and sweet. The smaller sizes are conveniently portable, and can be taken into the car and placed under the seat or hung on the hooks above, so as to be always at hand. The advantages afforded by an article so universally useful as this will readily suggest themselves to any person.

The cut at the head of this page represents the larger sizes. The smaller ones have a cross handle, like the common market basket, convenient for carrying on the arm.

The price is from $6 to $15, according to size.

## Figures and Tables

**Figure f1:**